# Smartphone virtual reality for pain management during pediatric burn care transition: study protocol for a randomized controlled trial

**DOI:** 10.1186/s13063-025-08860-4

**Published:** 2025-05-13

**Authors:** Megan Armstrong, Amber Price, Rebecca Coffey, Dana Noffsinger, Rajan K. Thakkar, Renata B. Fabia, Jonathan I. Groner, Samuel Mandell, Ai Ni, Henry Xiang

**Affiliations:** 1 Center for Pediatric Trauma Research, The Abigail Wexner Research Institute at Nationwide Children’s Hospital, 700 Children’s Drive, Columbus, OH 43205 USA; 2 Center for Injury Research and Policy, The Abigail Wexner Research Institute at Nationwide Children’s Hospital, 700 Children’s Drive, Columbus, OH 43205 USA; 3 Department of Surgery, University of Texas Southwestern Medical Center, 5323 Harry Hines Blvd, Dallas, TX 75390 USA; 4 Burn Program, 5200 Harry Hines Blvd, Parkland HealthDallas, TX 75235 USA; 5 Department of Pediatric Surgery, Nationwide Children’s Hospital, 700 Children’s Drive, Columbus, OH 43205 USA; 6 Department of Pediatrics, The Ohio State University, 370 West 9 th Avenue, Columbus, OH 43210 USA; 7 Division of Biostatistics, The Ohio State University College of Public Health, Columbus, OH USA

**Keywords:** Burn, Virtual reality, Children, Home care, Distraction

## Abstract

**Background:**

Burn injuries severe enough to result in emergency department visits are a large burden for US children. Treatment for these injuries often involves daily dressing changes at home, which can be very painful and anxiety-inducing. This trial aims to evaluate the efficacy of a virtual reality therapeutic for pain and anxiety alleviation during repeated at-home burn dressing changes among pediatric patients.

**Methods:**

Two hundred children with burn injuries requiring daily dressing changes will be recruited from two American Burn Association-verified burn centers in the USA for this randomized, controlled, two-arm clinical trial with a 1:1 allocation ratio. During each dressing change at home, the intervention group will play the Virtual Reality Pain Alleviation Therapeutic (VR-PAT) game and answer questions about their pain, anxiety state, pain medication usage, simulator sickness symptoms, and experience playing the game. The control group will perform their dressing as usual without the VR-PAT and answer questions about their pain, anxiety state, and pain medication usage. The primary outcome is the difference in self-reported pain and anxiety between the two groups over their week of dressing changes.

**Discussion:**

The transition of burn injury treatment from the medical center to the home can increase anxiety for children and their parents. Virtual reality is a promising digital technology that can improve wound care for these children. Findings from this trial will provide data on the efficacy of the VR-PAT for reducing self-reported pain and anxiety during daily home dressing changes for pediatric burn care. The results from this trial will serve as evidence for a large-scale implementation study.

**Trial registration:**

ClinicalTrials.gov NCT05673551. Registered on December 21, 2022.

## Administrative information

Note: the numbers in curly brackets in this protocol refer to SPIRIT checklist item numbers. The order of the items has been modified to group similar items (see http://www.equator-network.org/reporting-guidelines/spirit-2013-statement-defining-standard-protocol-items-for-clinical-trials/).

**Table:** 

Title {1}	Smartphone virtual reality for pain management during pediatric burn care transition: study protocol for a randomized controlled trial
Trial registration {2a and 2b}.	ClinicalTrials.gov: NCT05673551
Protocol version {3}	Version 3; February 22, 2024
Funding {4}	Agency for Healthcare Research and Quality (R01HS29183-01) https://www.ahrq.gov/ The funders had no role in study design, data collection and analysis, decision to publish, or preparation of the manuscript.
Author details {5a}	^1^ Center for Pediatric Trauma Research, The Abigail Wexner Research Institute at Nationwide Children's Hospital, 700 Children's Drive, Columbus, OH 43205, USA ^2^ Center for Injury Research and Policy, The Abigail Wexner Research Institute at Nationwide Children's Hospital, 700 Children's Drive, Columbus, OH 43205, USA ^3^ Department of Surgery, University of Texas Southwestern Medical Center, 5323 Harry Hines Blvd, Dallas, TX 75390 USA ^4^ Burn Program, Parkland Health, 5200 Harry Hines Blvd, Dallas, TX 75235, USA ^5^ Department of Pediatric Surgery, Nationwide Children's Hospital, 700 Children's Drive, Columbus, OH 43205, USA ^6^ Department of Pediatrics, The Ohio State University, 370 West 9 th Avenue, Columbus, OH 43210, USA ^7^ Division of Biostatistics, The Ohio State University College of Public Health, Columbus, Ohio, USA.
Name and contact information for the trial sponsor {5b}	Sheena Patel, MPH Sheena.Patel@ahrq.hhs.gov
Role of sponsor {5c}	The content is solely the responsibility of the authors and does not necessarily represent the official views of the Agency for Healthcare Research and Quality. The funder had no role in the study design; collection, management, analysis, and interpretation of data; writing of the report; and the decision to submit the report for publication, including whether they will have ultimate authority over any of these activities.

## Introduction

### Background and rationale {6a}

According to the Centers for Disease Control and Prevention, there were about 122.9 per 100,000 US emergency department (ED) visits for nonfatal burn injuries among children (0–18 years) in 2023 [1]. Over half of the pediatric burn injuries seen in US EDs are severe enough to merit referral to a burn center according to the USA and international guidelines [2, 3]. After discharge from burn care facilities, repeated burn dressing changes are often needed at home for 2–3 weeks. Pediatric patients have identified dressing changes as very painful, with opioid and anxiety medications often being prescribed [4, 5]. Furthermore, the pain experienced during burn dressing changes may cause distress to caregivers [6]. A painful experience can also serve as a stressor that significantly impacts patients’ post-injury health outcomes [7, 8]. The medical community in the USA is working to find the right balance between the risk of undertreating pain and causing unneeded suffering [4, 5] and the risk of over (or inappropriate) prescription of opioids [9]. Therefore, there is a pressing need to seek non-pharmacological interventions for effective pain management in pediatric burn wound care.

Pediatric clinicians widely utilize non-pharmacological pain management as a safe and affordable solution for procedural acute pain management [10, 11]. According to the Cognitive-Affective Model of Pain [12], pain perception demands cognitive attention. Thus, effective non-pharmacological pain management requires the interruption of the cognitive route from the origin to pain perception, redirecting a child’s attention resources away from the painful procedure. Virtual reality (VR) has been demonstrated to effectively decrease pain and distress in a variety of settings, among diverse populations [13, 14], and targeting a wide range of pain conditions (i.e., acute, procedural, and chronic) [15]. Recent meta-analyses and reviews of published studies in the past three decades have provided evidence that VR can effectively help patients reduce pain and anxiety across many settings [14–18]. However, prior studies have not investigated the feasibility and barriers of VR games for pain management during at-home burn dressing changes. Nearly all existing studies used computer-based VR equipment that is technologically and financially inaccessible to many patient families for everyday use in the home.

Our previous clinical studies have provided strong evidence about the efficacy of smartphone-based VR Pain Alleviation Therapeutics (VR-PAT) for significant pain reduction during burn dressing changes. In our randomized controlled trial (RCT) in the outpatient burn clinic, patients were randomly assigned to an active VR-PAT, passive VR-PAT, or standard care group [19]. Active VR-PAT significantly reduced observed and self-reported pain during burn dressing changes, while patients and caregivers reported satisfaction with the VR-PAT. In another pilot study, pediatric patients were randomly assigned to VR-PAT or control to examine the feasibility of VR-PAT as a pain alleviation tool during at-home dressing changes [20]. Children found the VR-PAT to be a helpful distraction during home dressing changes and reported it to be easy to implement. Children playing the VR-PAT reported consistent happiness and fun as the week went on and increased realism and engagement, indicating that repeated use does not diminish its efficacy.

In this proposed study, we will evaluate the effectiveness of a hands-free, portable, affordable, and actively engaging VR-PAT for pain and anxiety alleviation during repeated at-home burn dressing changes.

### Objectives {7}

The objective of this study is to evaluate the effectiveness of our smartphone VR-PAT as a pain distraction tool during repeated at-home burn dressing changes among 100 children (age 6–17 years, inclusive) with a burn injury in comparison to 100 children with a burn injury who do not use the VR-PAT.

Three specific aims will be pursued by recruiting a total of 200 pediatric burn patients (6–17 years) from two large burn centers:

- Aim 1: Evaluate the effectiveness of VR-PAT for pain management and opioid pain medication reduction during at-home burn care.
- *Hypothesis 1*: VR-PAT will induce clinically meaningful pain score intensity and opioid consumption (PIOC) reduction (>30%) during at-home burn care.
- Aim 2: Examine continuous engagement of patients and caregivers with VR-PAT during repeated at-home burn dressing changes.
- *Hypothesis 2*: Pediatric patients remain effectively engaged and interested in using the VR-PAT during repeated at-home burn dressing changes.
- Aim 3: Determine if there is a pain threshold for VR effectiveness.
- *Hypothesis 3*: VR-PAT is less effective for patients who have minor pain (Numeric Rating Scale (NRS) 1–2) than compared to moderate or severe pain.

### Trial design {8}

We will conduct an RCT with a 1:1 allocation ratio to either the *intervention group* (VR-PAT headset group) or the *control group* (no VR headset or game was provided). Pain, anxiety, and VR engagement (for those in the intervention group) will be assessed daily with each home dressing change for about 1 week.

## Methods: participants, interventions, and outcomes

### Study setting {9}

Participants will be recruited from American Burn Association-verified burn centers in the Midwest (Nationwide Children’s Hospital (NCH), Columbus, Ohio) and Southwest (Parkland Health, Dallas, TX). The list of study sites can be obtained on ClinicalTrials.gov or by contacting the corresponding author. The institutional review board (IRB) at NCH approved the study protocol under a single IRB agreement with the University of Texas Southwestern Medical Center.

### Eligibility criteria {10}

Eligibility will be assessed using the following criteria: (a) undergoing treatment for acute burn injury; (b) age 6–17 years, inclusive; (c) receiving their first outpatient clinic dressing change or being discharged from the ED or inpatient burn unit; (d) having a dressing that requires daily changes at home for about 5 days after their first outpatient appointment or discharge from the hospital; (e) patient and family caregivers can communicate (read and write) using English or Spanish; and (f) report an NRS pain score of ≥ 1 (NRS 0–10 with 10 being worst pain) from the most recent dressing change. To minimize the possibility of confounding variables, the following exclusion criteria will be applied: (a) any wounds that may interfere with study procedures (i.e., face burns); (b) vision, hearing, or cognitive/motor impairments preventing valid administration of study measures; (c) history of motion sickness, seizure disorder, dizziness, or migraine headaches precipitated by visual auras; (d) minors in foster care, prisoners, or those who are currently pregnant; (e) suspected child abuse; (f) unable to communicate in English or Spanish; and (g) families who do not have access to a VR compatible smartphone.

### Who will take informed consent? {26a}

Once patients are screened as eligible to participate, research study coordinators will approach the legal guardian and eligible child in the hospital (if inpatient) or Outpatient Burn Clinic for consent. Coordinators will ensure that the assenting child is awake, alert, and willing to be approached for research. Research staff will proceed with consent if both the patient and the patient’s guardian are interested in participating in the research after the study introduction by the care team. During the consent, the coordinator thoroughly ensures that the participant and guardian understand the research study, their role in participation, that participation is voluntary, would not affect treatment, and that they can withdraw at any time. The participants and guardians are informed of any risks associated with participation, and that participation in the study does not have any additional costs to their standard of care treatment. Once all questions are answered, the legal guardians give written consent, and the pediatric patient over 9 years of age gives written assent (per IRB guidelines).

### Additional consent provisions for collection and use of participant data and biological specimens {26b}

During the caregiver and participant’s informed consent conversation, the caregiver will be asked for permission to store the subject’s protected health information (PHI) and identifiable information for future IRB-approved research. The caregiver (parent/guardian) can opt out of this option, which will not affect their standard of care treatment. Biological specimens will not be collected for this study.

## Interventions

### Explanation for the choice of comparators {6b}

VR-PAT is a standalone mHealth tool that was developed at NCH and thoroughly tested in this patient population [19, 20]. It does not require an internet or Wi-Fi connection beyond downloading the app and consists of a lightweight VR viewer and an engaging Virtual River Cruise game played on a smartphone. Children play the virtual game by slightly tilting their heads without arm or hand movement, minimizing interference with the dressing change. A standard of care comparator was chosen as this study is conducted at home. We want this distraction tool to fit into the home setting in the future, so comparing it to what families would be doing otherwise was a critical aspect.

### Intervention description {11a}

The Virtual River Cruise game within the VR-PAT system allows a child to gently cruise on a boat and aim for the snow-blowing statues along the banks of the river. The statues emit snow if the child correctly aims at them, and a thermometer placed in the front of the boat shows decreased temperatures as more snowflakes are blown. As feedback to reinforce continued engagement, a scoreboard placed beside the thermometer will show children the number of statues they have activated. Additionally, as the temperature drops, snow and ice will start piling up on the boat and its surroundings, providing an enhanced “cooling” experience for pediatric burn patients. The directionally adaptive audio matches the progress of the game and the direction in which a child’s head is turning, further enhancing the immersive experience of VR-PAT during burn dressing changes. The active VR game can last indefinitely, so it can be used for the entire at-home dressing change with a charged battery without being interrupted. VR-PAT will be delivered by whoever is helping the child in the intervention group with their dressing change. Each participant in this group will complete one session for each required dressing change for about 1 week.

### Criteria for discontinuing or modifying allocated interventions {11b}

VR intervention group participants will be advised to discontinue using the headset during a dressing change if it causes motion sickness, headaches, nausea, or dizziness. Participants are welcome to try using the headset again for the next change but have the option of continuing the dressing changes and surveys without it or withdrawing from the study. Any adverse events from participation will be noted and reported on the subject surveys.

### Strategies to improve adherence to interventions {11c}

To improve study completion and participation, all subjects will receive a VR headset and an app download for their participation in the study. After returning the daily surveys, participants will receive $25 on a Greenphire ClinCard. Study team coordinators will encourage completion and adherence to the protocol by offering various options for follow-up. Surveys can be returned in person, by mail via a pre-paid envelope, or scanned and emailed to the coordinator. The study team will conduct calls before the follow-up appointment and up to three calls after to remind subjects to return surveys.

### Relevant concomitant care permitted or prohibited during the trial {11d}

The use of concomitant medications is allowed per the standard of care for treating the burn injury and pain management. Caregivers are requested to note the medication and the dosage on the caregiver survey report for each day they are used.

### Provisions for post-trial care {30}

Not applicable. No post-trial care is provided to study participants. No compensation is provided to participants who are harmed by participation.

### Outcomes {12}

#### Primary outcome measure

Change in self-reported and caregiver-reported worst pain, average pain, and time spent thinking about pain (NRS 0 (min)–10 (max)). These pain questions are assessed at each dressing change for about 1 week. Changes in pain will be compared between the intervention and control groups.

#### Secondary outcome measures

Change in self-reported anxiety (Spielberger State-Trait Anxiety Inventory for Children (STAI-CH) score 6 (low anxiety)–24 (high anxiety)). Anxiety is assessed at baseline (recruitment visit) and before each dressing change for about 1 week. The changes in anxiety will also be compared between the intervention and control groups.

Pain medication reduction. The caregiver will report pain medication usage (name and dosage) for about 1 week after each dressing change. The pain medication used will be compared between the intervention and control groups.

Average self-reported VR experience (NRS 0 (min)–10 (max) for the degree of realism, pleasure, and satisfaction with VR). For about 1 week, these VR experience questions are assessed at each dressing change for those in the intervention group.

### Participant timeline {13}

The participant timeline will consist of two visits and about 1 week of home dressing changes between them. The duration of participation is approximately 1 week after the study introduction. We anticipate participant recruitment to last 48 months. The timeline by intervention group and procedures is listed in Table 1 below.

**Table 1:** Survey procedures and timing of study visits

	**Visit 1** (Enrollment)	**Home dressing changes** (~ 1 week)	**Visit 2** (~ 7 days after enrollment)
Baseline survey (*n* = 1)	X		
Randomization	X		
VR-PAT download and headset distribution	Intervention group		Control group
Child questionnaire (*n* = 7)		X	
Caregiver questionnaire (*n* = 7)		X	
Medication questionnaire (*n* = 7)		X	
Follow-up survey (*n* = 1)			X
Incentive distribution (at survey return)			X

### Sample size {14}

The sample size was calculated for the primary outcome of the child-reported overall pain score. We consider a 30% reduction in the pain NRS score as a minimal clinically meaningful reduction in pain. According to our previous publication on pain reduction of VR-PAT among pediatric patients 6–17 years of age who were treated at our outpatient burn clinic [19], the pooled standard deviation (SD) of the child-reported overall pain visual analog scale (VAS) score was estimated. Aiming at detecting a 30% reduction of the pain score in the VR-PAT group compared with the control group at any of the first 7 days of follow-up, and assuming the same SD on these days, we will need 89 subjects per group to achieve 80% power and a two-sided type-I error of 0.01 at each day using a two-sample *t*-test. The type-I error rate is adjusted by Bonferroni correction for multiple comparisons. Assuming a 10% dropout rate, we will need 99 subjects per group. Based on this calculation, we plan to *n* = 100 subjects per group (*n* = 200 subjects total).

### Recruitment {15}

Participants will be recruited after their first dressing change in the Outpatient Burn Clinic or at discharge from the ED or inpatient burn unit. Patients of age will be initially screened daily through the electronic medical record system at each hospital for general eligibility. Once the treating clinician assesses the burn wound and determines the type of dressing needed, trained research staff will approach patients receiving a daily dressing to assess their pain status and smartphone availability. Study introductions are made during this in-person visit, and informed consent/assent is obtained from interested caregivers and patients. No recruitment materials will be used to advertise the study.

## Assignment of interventions: allocation

### Sequence generation {16a}

A random-block randomization scheme with a block size of four will allocate participants to either intervention or control at a 1:1 ratio without stratification. Each site had its own randomization scheme stored in its respective Data Access Group in REDCap to ensure that subjects within each arm were distributed evenly between each site.

### Concealment mechanism {16b}

Study participants are randomized into control and intervention groups after signing consent and completing the baseline survey. Research recruiters and subjects do not know the intervention assignment until the subject is randomized in REDCap.

### Implementation {16c}

The project manager generated the allocation sequences and uploaded them to the Randomization Module in REDCap. The research study team involved in recruitment will screen and enroll subjects into the study and assign them to intervention groups by using the randomize button within REDCap, which only reveals the intervention group for the current participant. The clinical care team will determine the method of care required for the patient to confirm if the subject will qualify for daily dressing changes. These decisions will be made based on treatment needs without research influence.

## Assignment of interventions: blinding

### Who will be blinded {17a}

We cannot blind the participants, caregivers, or research team from the assigned intervention since one group will use the VR headset and one will not. The study biostatistician will be blinded to the intervention group while performing analyses on the primary outcome. The data will be blinded by removing labels such as “VR” and “Control” and will be replaced with unidentifiable letters (i.e., “A” and “B”). The biostatistician will need to become unblinded for secondary outcomes as those in the intervention group will be the only ones answering the VR experience questions.

### Procedure for unblinding if needed {17b}

The biostatistician will be unblinded for full analyses by providing the full dataset with complete data labels and VR questions. This unblinding will occur after the primary outcome analysis has been completed.

## Data collection and management

### Plans for assessment and collection of outcomes {18a}

#### Content and protocol for the control group

Participants in the control group are given a folder of daily surveys and instructed to complete their standard wound care as instructed by their care team. Then, they complete the provided daily surveys for both the child and caregiver, indicating the date and dressing change number for each day.

#### Child surveys (self-reported)

All participants (control and intervention group) will self-report anxiety prior to the dressing change using the STAI-CH scale with ratings from not at all to very much. After the dressing change is completed, the child will answer the remaining survey questions assessing overall pain score (NRS, 0–10), worst pain score (NRS, 0–10), and time spent thinking about pain (NRS, 0–10). The survey is completed once daily alongside dressing changes for 7 days until the patient returns for their follow-up appointment. Survey questions remain the same for each daily dressing change.

#### Guardian surveys (proxy report)

Caregivers (control and intervention group) of the participating child conducting the dressing change are asked to report their observation of the child’s pain after wound care (NRS, 0–10). Caregivers are also asked to document any opioid or pain medication given (name, brand, and dose) and are given the opportunity to share anything of note for that dressing change. The survey remains the same for each day of wound care and is to be completed once daily after the child surveys.

#### Content and protocol for the intervention (VR) group

Participants in the intervention (VR-PAT) group are given a folder similar to the control group with daily surveys, with the addition of download instructions for the VR-PAT smartphone application and a VR headset best suited for the participating child. The child is asked to wear and play the approved VR game during the dressing change, and then both the child and caregiver are instructed to complete the daily surveys after wound care.

#### VR-PAT child surveys (self-reported)

In addition to self-reporting anxiety STAI-CH survey questions prior to wound care and assessing their overall pain score (NRS, 0–10), children are asked about their experience with the VR game (degree of realism, fun, engagement, and satisfaction) and pain perception while playing the VR game (NRS, 0–10). Participants are also asked to report any simulator sickness associated with the VR game.

#### VR-PAT guardian surveys (proxy report)

Primary caregivers will complete the same pain assessment (NRS, 0–10) and medication documentation as the control group, with the addition of questions regarding the VR-PAT use. Caregivers will note the amount of time the child played, willingness to participate, number of wound care interruptions, ease of use, and helpfulness of the device. If any other details about the dressing change would like to be shared, a comment section is provided for notes.

### Plans to promote participant retention and complete follow-up {18b}

Based on our pilot study, we do anticipate losing some participants to follow-up. We will attempt to promote retention by emphasizing the importance of returning surveys after their week of home dressing changes at the time of recruitment. The recruiter will also ask the legal guardian for a phone number where they can receive reminder calls. Participants in the VR group will be asked to download the app at the time of recruitment; however, if they choose not to, they may download it at home and are provided with contact information for the research team and receive a check-in phone call on the next business day following recruitment to ensure there were no technological issues. All participants will receive a reminder call before their next clinic appointment (or at the 7-day mark if they are not returning to the clinic). Additional reminder calls will be made once per week for up to 3 weeks to encourage the return of surveys. We will also allow participants to return surveys in person at a clinic appointment, by taking a photo and emailing them to an email address created specifically for this project, or by mailing them in a provided prepaid, self-addressed envelope. Participants who return surveys, thus completing all study procedures, will receive an incentive of $25 on a Greenphire ClinCard.

### Data management {19}

Data from the baseline survey, child and caregiver questionnaires, and follow-up survey will be independently entered and stored electronically in a secure REDCap database hosted on the NCH servers by trained research staff. Only researchers listed on the approved IRB will be granted access. Once data have been collected and entered, another team member or PI will verify the records to ensure the information is complete. Research staff from each site will only have access to participant data within their Data Access Group, except for the study coordinator and the contact PI, who will have access to the full data set. Each research participant is assigned a unique Study ID. The master file that links the participants’ names to their Study IDs will be maintained only in a secure file folder in a password-protected computer at the Research Institute at NCH and Parkland Health. All measurement and randomization procedures will only use Study IDs to record data without participants’ names attached.

Any technical-related beta feedback provided from iPhone users through the TestFlight app will be received via NCH e-mail, and all identifiers will be removed before being saved in a secure file folder on a password-protected computer at the Research Institute at NCH. De-identified technical feedback will be forwarded to the VR developers (listed on the IRB application) to assist with improving the app for future studies.

### Confidentiality {27}

Prior to enrollment, screening spreadsheets and other electronic databases to access patient charts will be kept in a secure file folder on password-protected computers in the Research Institute at NCH and at Parkland Health. Only researchers on the IRB will have access to the secure folder where the file is located.

Eligible subject information will be entered in a locked research office on a hospital-approved password-protected computer. Enrolled subjects will be assigned a study ID number. The PI and limited trained research staff will maintain all components of the research record containing participants’ identifying information.

Hard copies and information collected during study enrollment are kept in a secure locked file cabinet in a locked office, with only study staff having access. Publication will not identify subjects or contain information leading to identification. The study team has no plans to contact the subjects after their study participation or to share PHI with anyone outside the study team.

### Plans for collection, laboratory evaluation, and storage of biological specimens for genetic or molecular analysis in this trial/future use {33}

Not applicable. No biological specimens will be collected.

## Statistical methods

### Statistical methods for primary and secondary outcomes {20a}

#### Aim 1

In the primary analysis, the child-reported overall pain NRS score will be compared between the VR-PAT and control groups during each of the first 7 days of follow-up using a two-sample *t*-test. The *p* values will be adjusted by Bonferroni correction to account for multiple comparisons and then compared to the critical value of 0.05 to determine statistical significance. In the secondary analysis, child-reported worst pain score, parent-observed overall and worst pain scores, child engagement and experience with VR, caregiver report of VR use, and adverse effects related to engagement with VR will be compared between the two study groups using a two-sample *t*-test (for continuous variables) and chi-squared test (for categorical variables). To estimate the overall difference in the child-reported overall pain score between the two study groups over the 7 days of follow-up, a linear mixed effect model with random intercept accounting for the correlation among repeated measures of pain scores will be fit. The dependent variable of the model will be child-reported overall pain score. The independent variables of the model will contain the study group, time (follow-up days), and any potential confounders deemed unbalanced between the study groups. The coefficient of the study group is the estimated overall difference in the pain score. To further explore whether the trend of child-reported overall pain score over the 7 days differs between study groups, the same linear mixed effect model as above will be fit with the addition of interaction between the study group and time. The coefficient of the interaction term estimates the difference in the trend of pain scores over time.

#### Composite pain score and opioid consumption (PIOC) score

Because opioids and other pain medications could also influence the pain score (interdependence), and to overcome key statistical challenges such as mass significance and increased risk of type 1 error, other researchers suggested a composite pain and opioid consumption (PIOC) score for analgesic clinical studies. We will apply the novel approach of this integrated outcome measurement by including longitudinally measured PIOC score over the 7-day follow-up period. The area under the curve for a child-reported overall pain (AUC-NRS) and morphine equivalent total dose of pain medications (AUC-OC) would be calculated by the trapezoidal rule. The trapezoidal rule estimates the AUC by summarizing the trapezoid areas under the graph between each pair of consecutive observations and then ranks AUC-NRS and AUC-OC for both VR and control groups. PIOC is the summation of the deviations from the mean ranks for both parameters and equals − 200% to + 200% for each patient. Effect size could then be expressed as the probability of having a better (lower) PIOC score with treatment compared to the control group. The *U* value from the Mann–Whitney *U* test will be used to calculate a “probability” (*P*′). The *P*′ could be applied in the estimation of a generalized odds ratio, which provides the effect size, including 95% CI. The generalized odds ratio can be interpreted as the probability of a randomly chosen patient in the intervention VR group having better ratings of the composite analgesic outcome than a randomly chosen patient in the control group. To the best of our knowledge, based on our review of the literature on VR studies, this innovative PIOC approach has not been used by other researchers in the assessment of the effectiveness of VR on pain score and opioid medication reduction. We have successfully applied this innovative pain outcome statistical test approach in our feasibility RCT study among 24 pediatric burn patients [20].

#### Aim 2

In the primary analysis, the mean child-reported VR engagement score in the VR-PAT group will be calculated along with a 95% confidence interval for each of the seven follow-up days. The intention-to-treat principle will again be used in the primary analysis by accounting for all subjects in the VR-PAT group in the calculation regardless of whether they actually use the VR device during dressing changes. Sensitivity analysis will also be conducted by imputing missing scores due to dropout and then recalculating the mean scores and their 95% confidence intervals. In the secondary analysis, the mean child-reported fun score and mean child-reported realism score will be calculated along with 95% confidence intervals for each of the 7 days. In addition, a linear mixed effect model with random intercept, dependent variable of child-reported VR engagement score, and independent variable of time will be fit to assess the trend of engagement score over the seven follow-up days. The coefficient of time will reveal the direction and magnitude of the trend. The same linear mixed effect models will be fit for child-reported fun scores and mean child-reported realism scores to assess their trends over time.

### Interim analyses {21b}

Not applicable. No interim analyses were planned.

### Methods for additional analyses (e.g., subgroup analyses) {20b}

Survey questions not listed as primary or secondary outcomes will be compared between the two study groups at each of the 7 days of follow-up. Subgroup analyses will be conducted by performing the same analyses as described above separately by sex and age (6–9 vs. 10–14, 15–17 years of age).

### Methods in analysis to handle protocol non-adherence and any statistical methods to handle missing data {20c}

The intention-to-treat principle will be used in the primary analysis, in that subjects will be analyzed according to their assigned study group regardless of whether they used the VR-PAT or in the control group during dressing changes. Subjects that drop out of the study will be excluded from analyses after their dropout date. As a sensitivity analysis, the missing pain scores due to dropout will be imputed by multiple imputations with chained equations, and two-sample *t*-tests will analyze the imputed dataset. For Aim 2, sensitivity analysis will be conducted by imputing missing scores due to dropout and then recalculating the mean scores and their 95% confidence intervals.

### Plans to give access to the full protocol, participant level data, and statistical code {31c}

Public access to the full protocol and statistical code is available upon reasonable request to the corresponding author. De-identified research data will be made available upon reasonable request to the Data Trust & Value Committee at Nationwide Children’s Hospital via e-mail at datatrustcmt@nationwidechildrens.org.

## Oversight and monitoring

### Composition of the coordinating centre and trial steering committee {5d}

This trial will be overseen and coordinated by the contact PI, two site PIs, and a project manager. Each site has at least one study coordinator to assist with the screening, recruitment, consent, and data collection. The site PIs monitor the research activities at their respective institutions, while the project manager and the contact PI monitor research progress study-wide. The contact PI and project manager meet weekly to discuss the project, including recruitment updates, challenges, and any reported adverse events. The project manager has monthly meetings with the study coordinators and weekly emails with recruitment updates. The full study team meets quarterly. There is no specific steering committee for this study.

### Composition of the data monitoring committee, its role and reporting structure {21a}

The NCH IRB did not require a data monitoring committee (DMC) as this is a minimal-risk study. The contact PI and the site PIs will be responsible for all research aspects of this study, including ensuring informed consent/assent policies are followed, keeping the study IRB compliant, and monitoring the data quality. The contact PI and site PIs will review all adverse events (AEs) and serious adverse events (SAEs). They will also ensure all AEs, SAEs, and protocol deviations are reported to the IRB and sponsor as per institutional requirements.

### Adverse event reporting and harms {22}

Participants in the intervention group will be asked to report at each dressing change to answer the question, “Did the game make you feel not well? If yes, please explain.” Study coordinators will carefully review collected data for any adverse events reported during the study intervention and immediately report this information to the project manager. The PIs will determine how serious the event was, whether it was related, and if it was unexpected. All SAEs will be reported immediately to the sponsor and IRB. At recruitment, participants and caregivers will be informed of what simulator sickness symptoms to be aware of and advised that if they occur, children can remove the headset and continue the dressing change without it. They can then choose whether to try again at the next dressing change or to continue their week of wound care without the headset. All participants will also receive a direct phone number for a study coordinator at each site to report adverse events.

### Frequency and plans for auditing trial conduct {23}

The contact PI and project manager will review this study weekly to monitor recruitment goals, participant retention, and any technology issues. As this was deemed a minimal-risk study, the NCH IRB will perform a study check every 2 years.

### Plans for communicating important protocol amendments to relevant parties (e.g., trial participants, ethical committees) {25}

We do not anticipate any important protocol changes, particularly those related to eligibility criteria or analytic plans. If any should occur, these modifications will be submitted to the IRBs responsible for approval before implementation. PIs and study coordinators will be informed via email during the regularly scheduled meetings that this modification will be submitted to the IRB, and they will be notified again via email when the IRB approves it. The ClinicalTrials.gov registration will also be updated as appropriate. Participants will only be notified of protocol changes if they directly affect their eligibility for participation.

### Dissemination plans {31a}

The dissemination efforts for the results from this trial will have two main components. The first will be the creation of a VR-PAT intervention implementation toolkit. Through this toolkit, we intend to inform potential new users and adopters about the VR-PAT intervention and our experiences implementing it. To facilitate the spread of the intervention, we will provide tools, guidelines, and tips for implementing VR-PAT based on the lessons learned and on what we found effective and useful in our implementation efforts.

The second method will be through peer-reviewed journal publications, national conference presentations, and ClinicalTrials.gov updates. We plan to submit one to two manuscripts in the field of pediatric trauma research or medical extended reality to peer-reviewed journals every year of the funding period. Additionally, we plan to present at one national conference each year to reach a broad audience of clinicians, researchers, and medical extended reality professionals. We hope that our publications and conference presentations will inform the medical and research community about our trial and results and encourage implementation at their institutions. Finally, we will use internal media relations and translational research teams to generate plain language summaries of findings that will be disseminated through press releases and social media posts.

## Discussion

Building on our pilot studies using VR-PAT for pediatric burn care, the protocol has generally been implemented well at our study sites. We have encountered slower-than-anticipated recruitment as changes in pediatric burn care have led to more long-term dressing usage than anticipated. According to a global survey of burn experts published in 2021, some of the most important characteristics of an ideal burn dressing were pain-free changes and requiring fewer changes [21]. The responses in this survey are consistent with the wound care trends we see in this ongoing study. We continue to monitor recruitment monthly and believe we are still on track to achieve our recruitment goals, but possibly not as early as originally planned. Our second challenge has been hosting the VR-PAT on personal cell phones. The application is compatible with both Android and iPhone platforms. However, smartphone security updates can interfere with downloading the app, particularly for iPhone users. The VR-PAT developer has been closely involved in the project and works quickly to adapt to changes. We have had small pauses in recruitment for iPhone users, which has not impacted our overall recruitment thus far.

Assuming this study’s successful completion, future research directions include a full-scale dissemination and implementation study. This would involve a rollout to American Burn Association-verified burn centers in the USA. We will also prioritize the Principles for Digital Development in designing our future dissemination study [22]. Specifically, one fundamental principle for this dissemination work will be building for sustainability. One consideration will be the long-term cost of ownership, which includes the possibility of insurance reimbursement. The RelieVRx [23] is a US Food and Drug Administration-authorized VR treatment that is recognized by a reimbursement code, paving the way for future VR devices. The efficacy data expected from this large, multisite clinical trial is essential before this next step.

In summary, pain and anxiety management during at-burn dressing care faces many challenges. Non-pharmacological pain management is needed as the nation is still fighting the opioid crisis and seeks innovative ways to reduce opioid pain medications. Smartphone VR apps offer attractive pain and anxiety management, particularly for children and adolescents who are familiar with gaming technologies. This study will provide evidence of the effectiveness and challenges of using smartphone VR for at-home pediatric burn care.

### Trial status

Protocol Version 3 (2/22/2024). Recruitment began on January 16, 2023; completion is expected by April 30, 2027.

## Data Availability

The datasets generated and/or analyzed during the current study are not publicly available due to them containing private health information (PHI) but are available on reasonable request. To request access to the de-identified research data, please contact the Data Trust & Value Committee at Nationwide Children’s Hospital via e-mail at datatrustcmt@nationwidechildrens.org. Deidentified data will be provided via a Microsoft Excel file.

## Abbreviations

AE: Adverse event
AUC: Area under the curve
DMC: Data Monitoring Committee
ED: Emergency department
IRB: Institutional Review Board
NCH: Nationwide Children’s Hospital
NRS: Numeric Rating Scale
PHI: Private Health Information
PIOC: Pain Score Intensity and Opioid Consumption
RCT: Randomized controlled trial
SAE: Serious adverse event
SD: Standard deviation
STAI-CH: Spielberger State-Trait Anxiety Inventory for Children
VAS: Visual analog scale
VR: Virtual reality
VR-PAT: VR-based Pain Alleviation Therapeutics
